# Supplementary material for: A general fruit acid chelation route for eco-friendly and ambient 3D printing of metals
Source: Nat Commun. 2022 Mar 7;13:104. doi: 10.1038/s41467-021-27730-6 (PMC8901924; doi:10.1038/s41467-021-27730-6)
Supplement: Supplementary file 1 — Supplementary Information [file 41467_2021_27730_MOESM1_ESM.pdf]

Supplementary Information for

A General Fruit Acid Chelation Route for Eco-friendly and Ambient 3D Printing of Metals

Soo Young Cho, Dong Hae Ho, Yoon Young Choi, Soomook Lim, Sungjoo Lee, Ji Won Suk, Sae Byeok Jo* & Jeong Ho Cho*

*Correspondence and requests for materials should be addressed to S.B.J. (e-mail: eos0523@gmail.com) or J.H.C. (e-mail: jhcho94@yonsei.ac.kr)

This PDF file includes:

Supplementary Fig. 1 to 23

Supplementary Table 1 to 2

Supplementary References

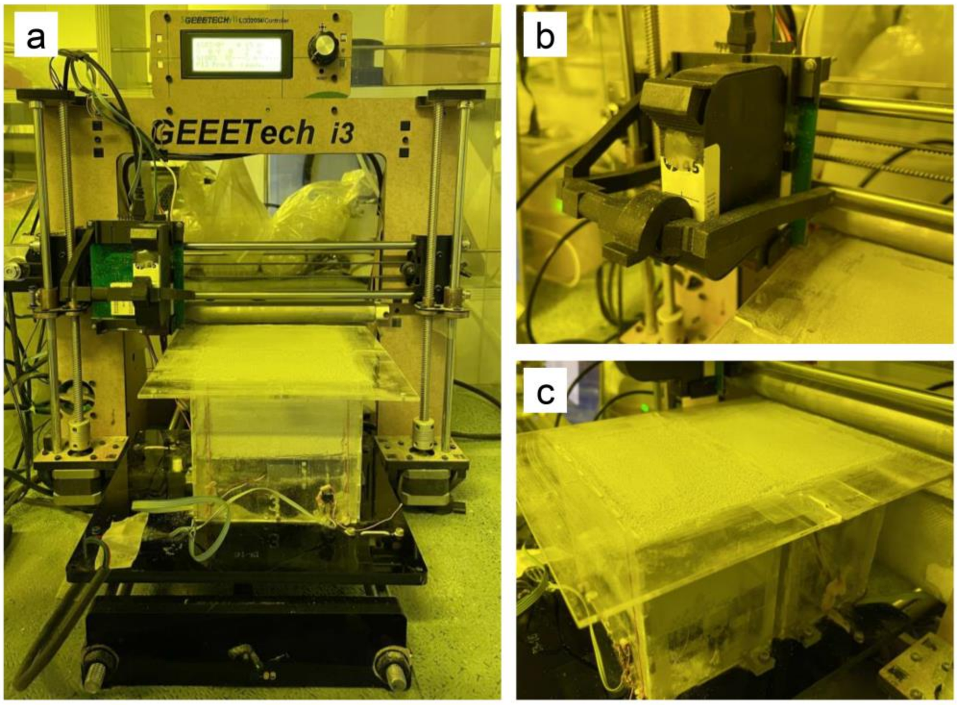

Supplementary Fig. 1. Photographic images of **a** binder jetting 3D printer, **b** inkjet cartridge and roller, and **c** powder gantry boxes.

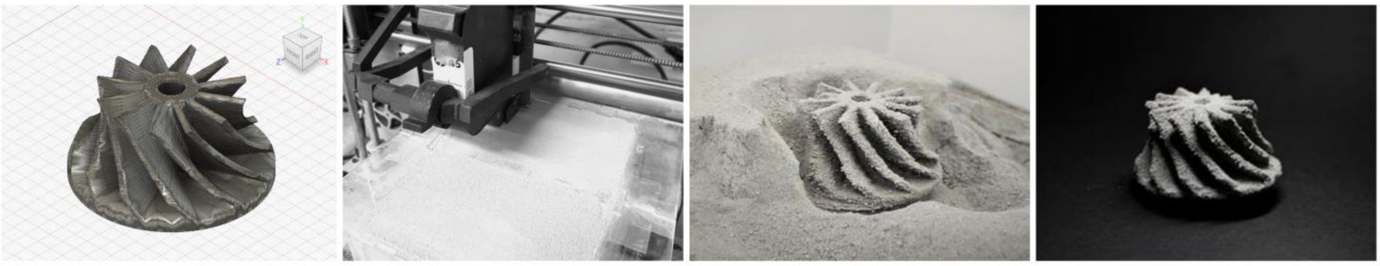

Supplementary Fig. 2. Procedure of BJM3DP using Al powder.

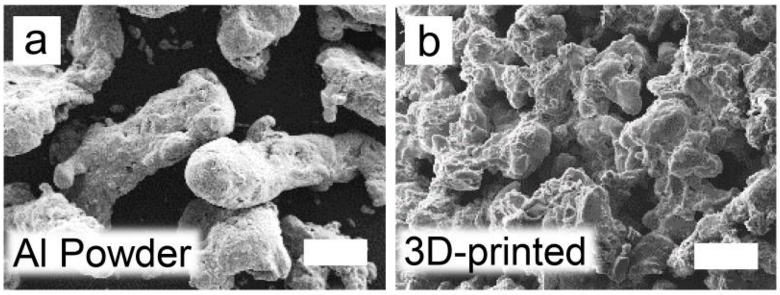

Supplementary Fig. 3. SEM images of pristine Al powder (left) and 3D-printed Al object (right) (scale bar: 100 μm).

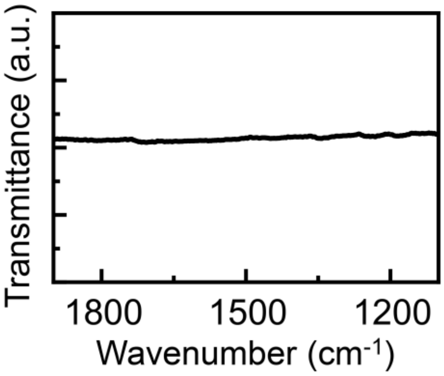

Supplementary Fig. 4. FT-IR spectrum of pristine Al powder.

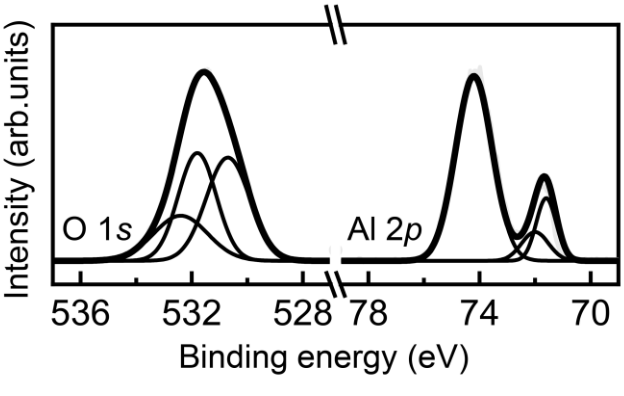

Supplementary Fig. 5. Al 2*p* and O 1*s* XPS spectra of pristine Al powder.

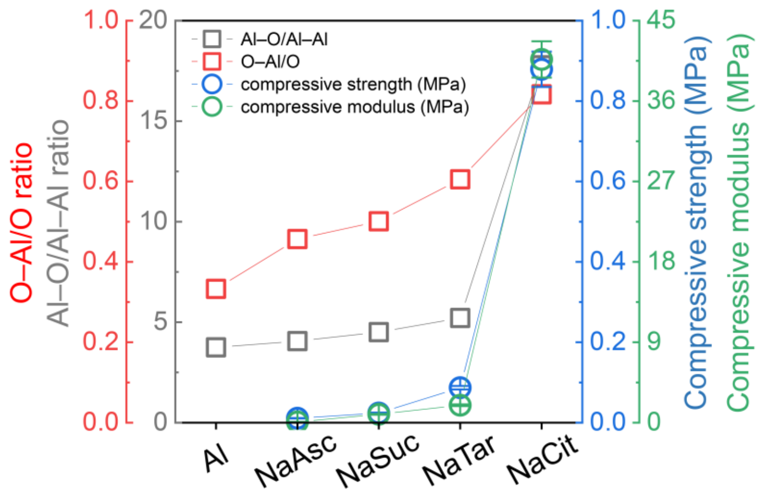

Supplementary Fig. 6. O–Al/O atomic ratio (from O 1*s* spectra), Al–O/Al–Al atomic ratio (from Al 2*p* spectra), compressive strength, and compressive modulus of object 3D-printed using different chelators having different numbers of coordination groups. Data for compressive strength and modulus of NaCit are presented as mean values ± standard deviations.

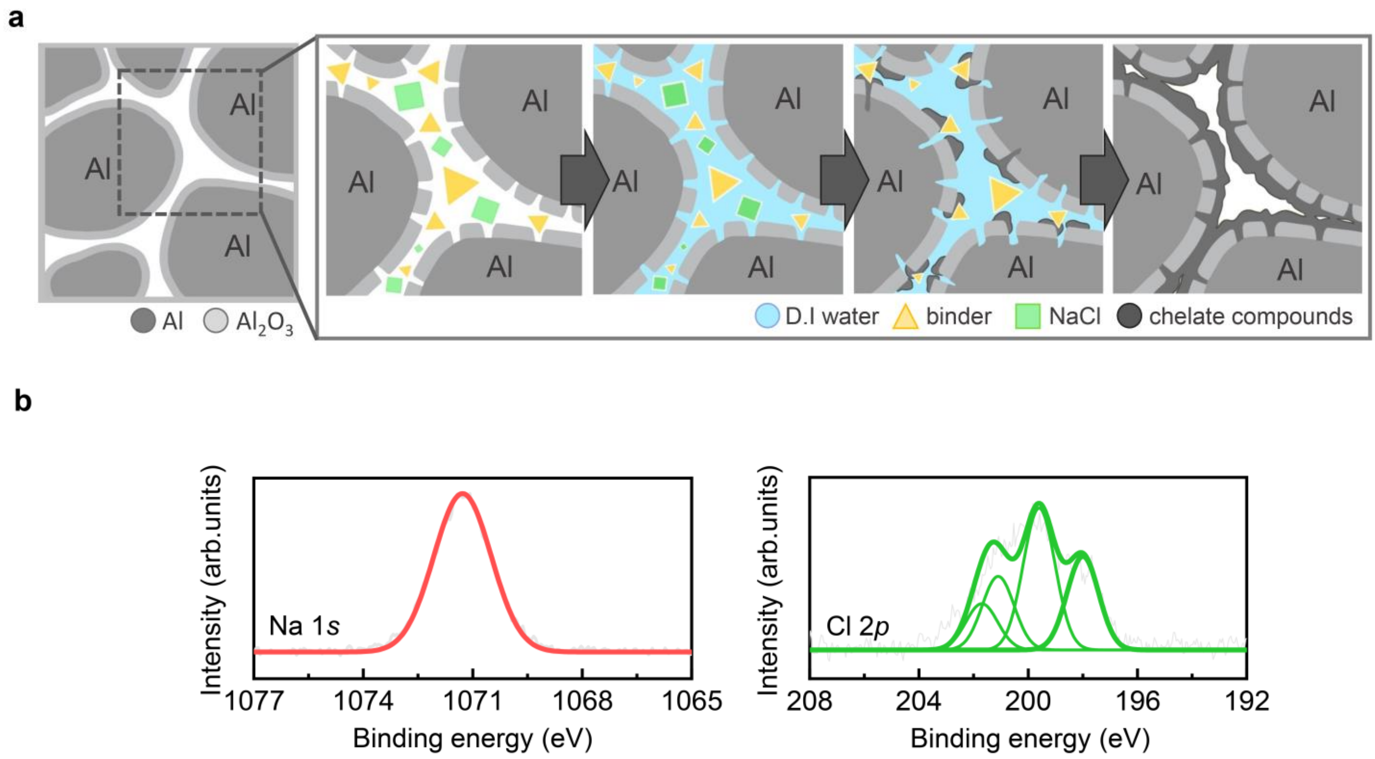

Supplementary Fig. 7. a Schematic illustration of metal chelation in presence of NaCl additive. **b** XPS spectra for Na 1*s* and Cl 2*p* of the printed Al object. After the process is completed, Cl^- ions mostly end up back in the form of NaCl with a trace amount of $AlCl_3$ left in the system.

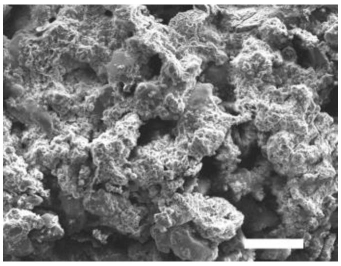

Supplementary Fig. 8. SEM image of Al object 3D-printed using NaCit chelator and NaCl additive (scale bar: 100 μm).

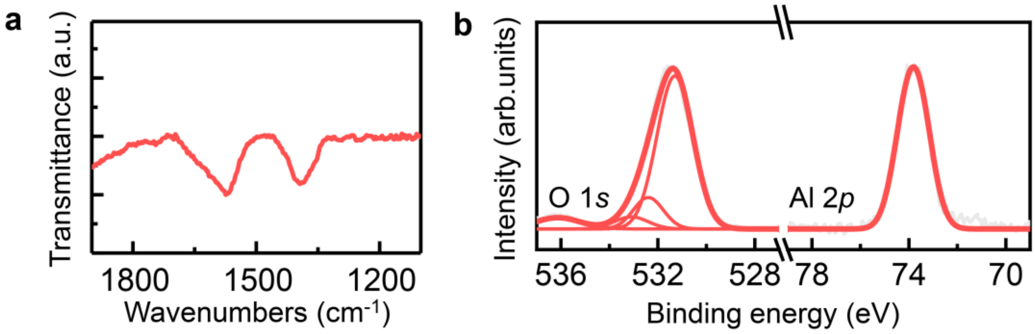

Supplementary Fig. 9. a FT-IR spectrum and **b** Al 2*p* and O 1*s* XPS spectra of Al object 3D-printed using NaCit chelator and NaCl additive. In the FT-IR spectra, the vibrational frequency difference (Δv) between asymmetric (v_{as}) and symmetric (v_s) stretching vibrations of COO^- reflects the change of molecular mass upon the reaction of aluminum and COO^-, and therefore the change in Δv for different chelating reactions can represent the degree of chelation. With NaCl additive, the Δv was decreased from 198 cm^{-1} (only NaCit) to 172 cm^{-1}, which is a clear sign of increased degree of chelation. v and Δv values for different systems are summarized in the **Supplementary Table 1**.

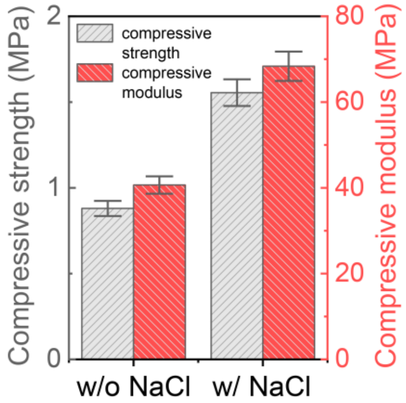

Supplementary Fig. 10. Comparison of compressive strength and compressive modulus of NaCl-additive-free 3D-printed object with those of NaCl-additive-treated 3D-printed Al object. Data are presented as mean values ± standard deviations.

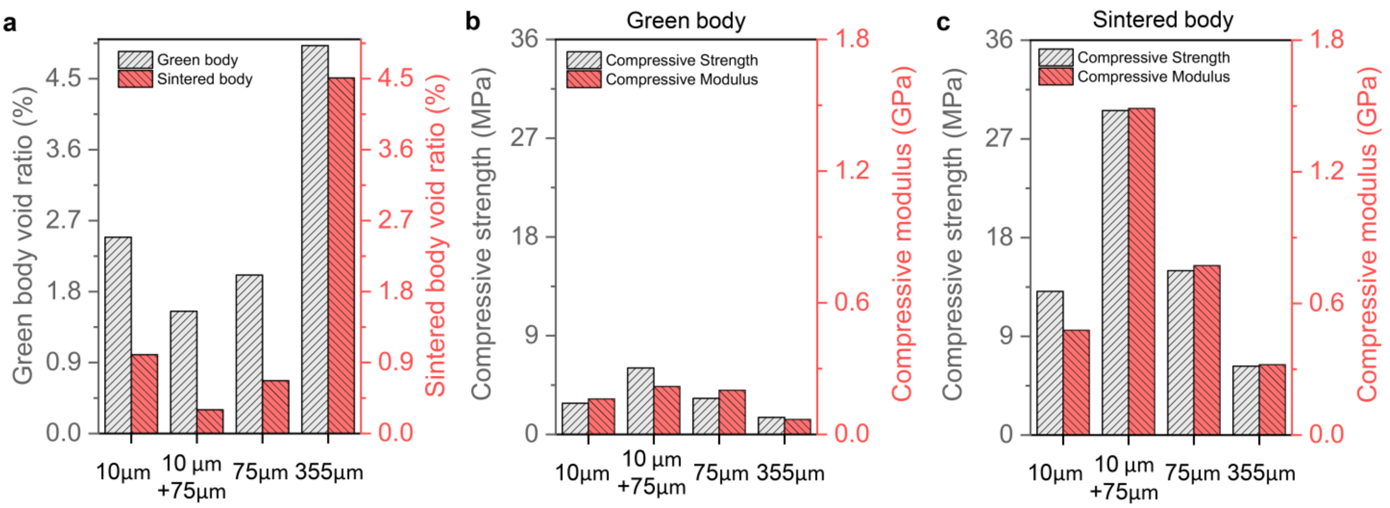

Supplementary Fig. 11. **a** The defect volume ratio, and mechanical strengths of **b** green bodies and **c** sintered bodies based on Al particles with different size distributions. An optimized NaCit/NaCl-based chelation route is adopted for all cases. The defect volume ratio of printed objects was acquired by using 3D computed tomography (μCT, Nikon XTH 320) with the voxel size of 1 μm at X-ray beam energy of 210 kV. Voxel analysis were performed by using VGSTUDIO MAX (Volume Graphics Pte. Ltd).

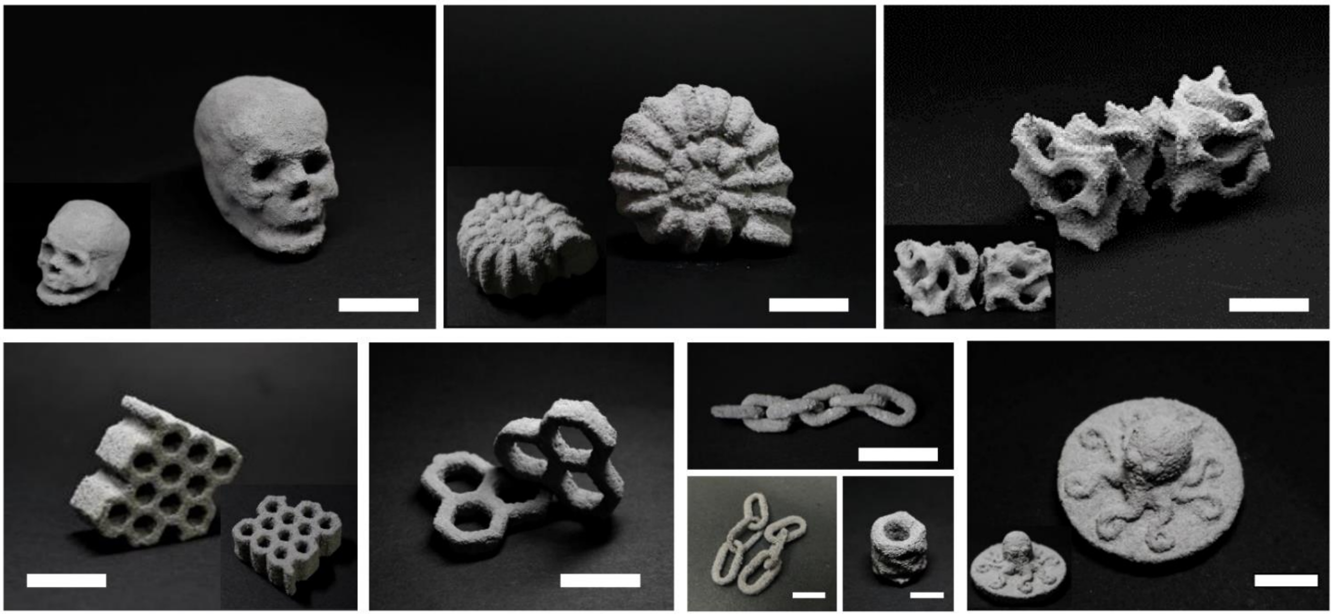

Supplementary Fig. 12. Photographic images of 3D-printed Al objects having different shapes (scale bar: 10 mm).

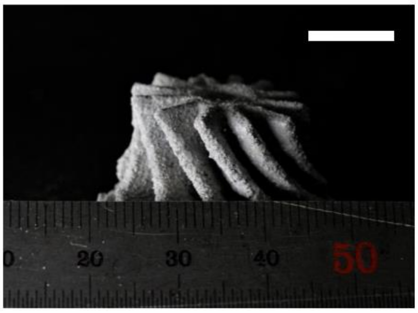

Supplementary Fig. 13. Photographic images of 3D-printed Al impeller having vane thickness of 1 mm (scale bar: 10 mm).

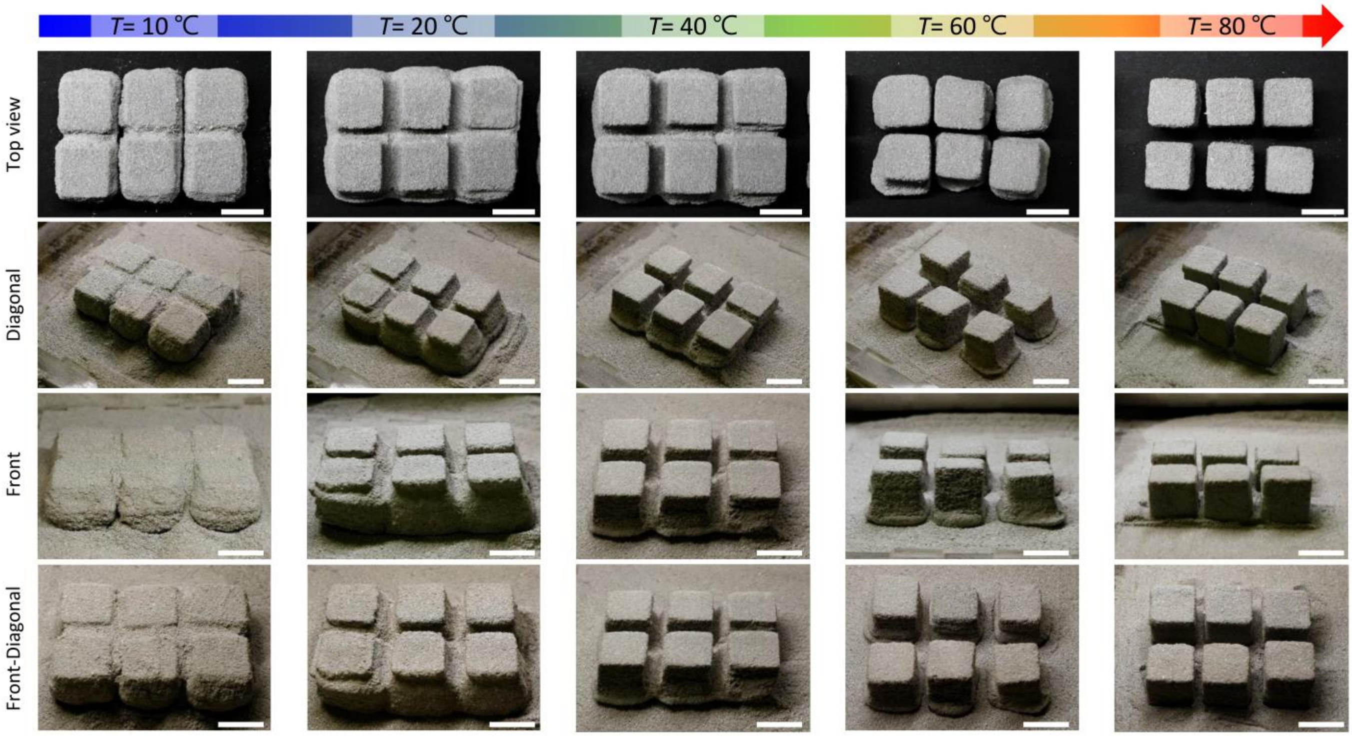

Supplementary Fig. 14. Demonstration of the minimization process for bleeding effects by using different initial printing conditions. The temperature of builder was gradually increased from 10 ºC to 80 ºC while the nozzle temperature was kept at 70 ºC. The scale bar on the image is 10 mm.

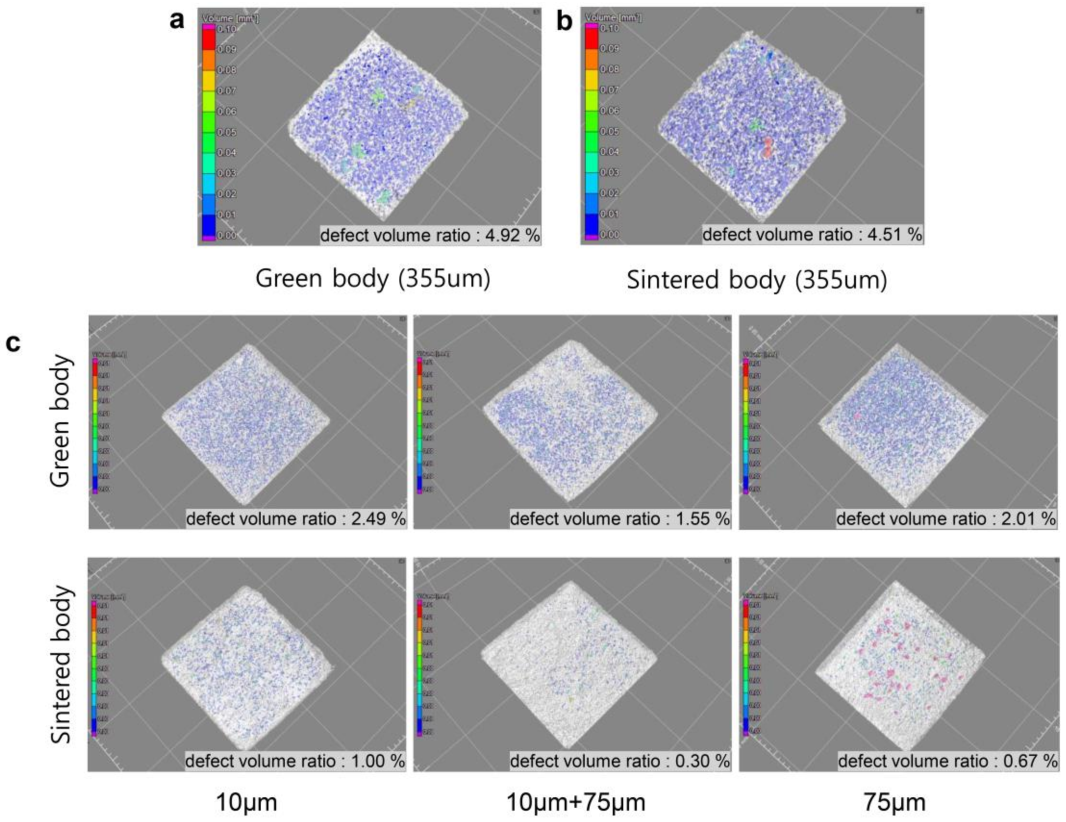

Supplementary Fig. 15. Rendered images and void ratios from μCT analyses for **a** green body and **b** sintered body based on 355 μm Al powders. **c** Rendered images and void ratios from μCT analyses for green and sintered bodies based on metal particles with various size distributions.

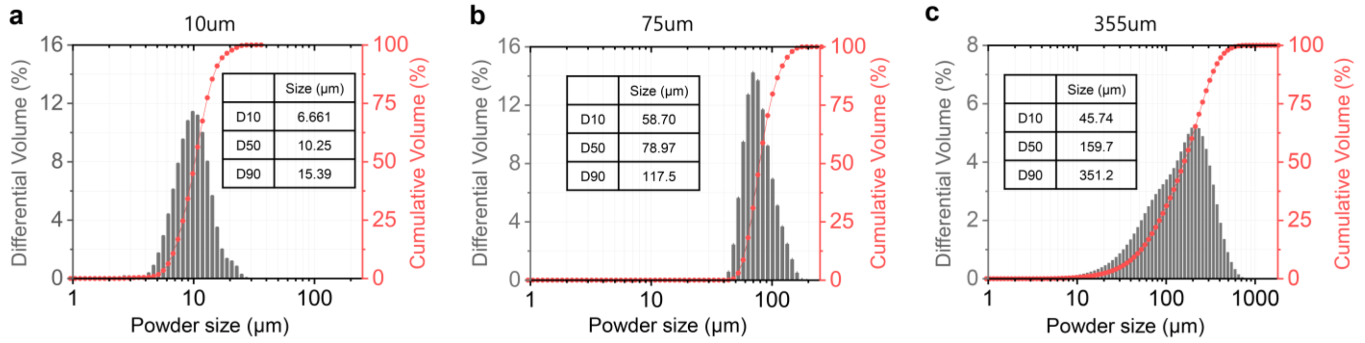

Supplementary Fig. 16. Powder size distribution of **a** 10 μm, **b** 75 μm and **c** 355 μm powders. The ISO 13320 laser diffraction method (Beckman Coulter LS-13-320) was used for the analysis.

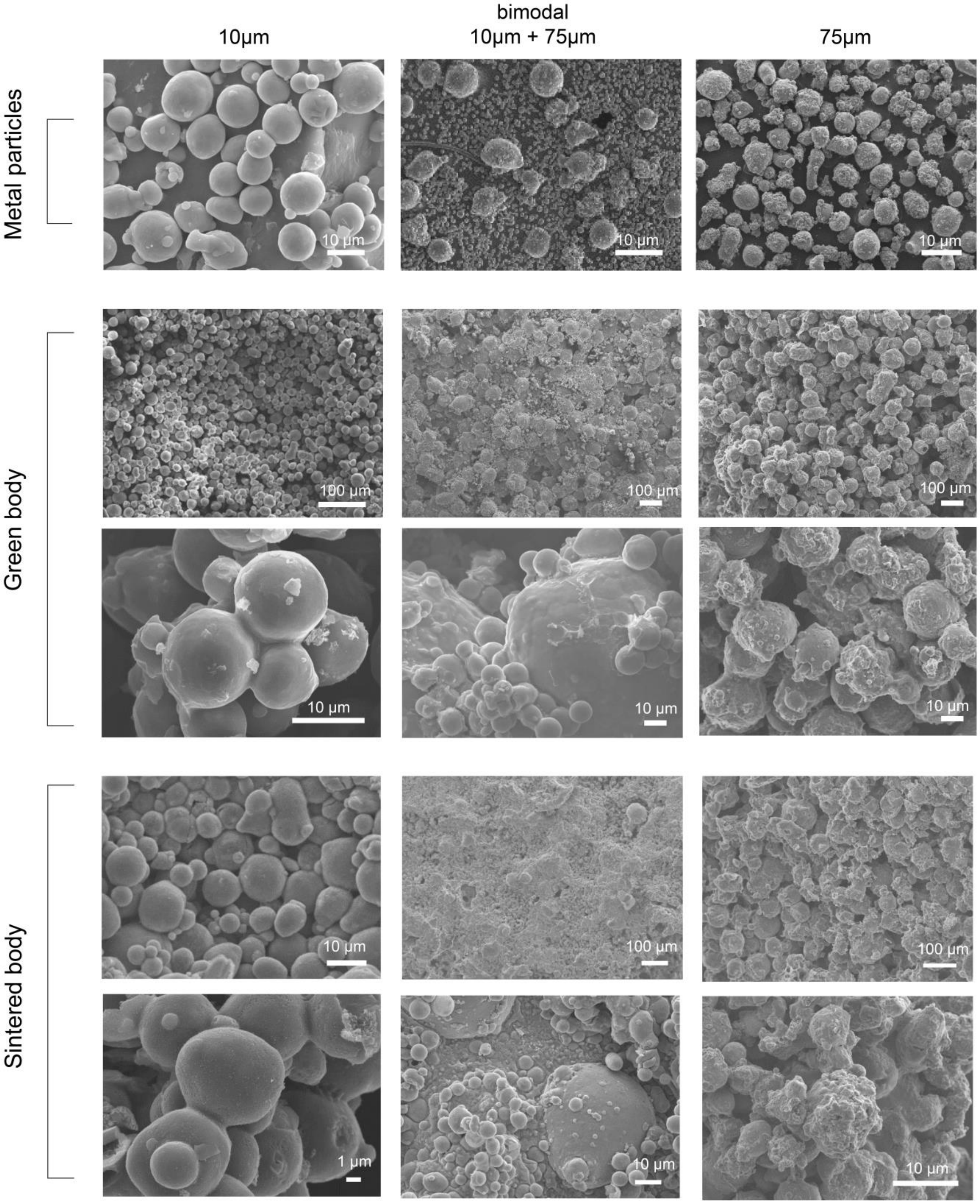

Supplementary Fig. 17. SEM images of metal particles, green body and sintered body used for further study, depending on the size and composition distributions of the particles.

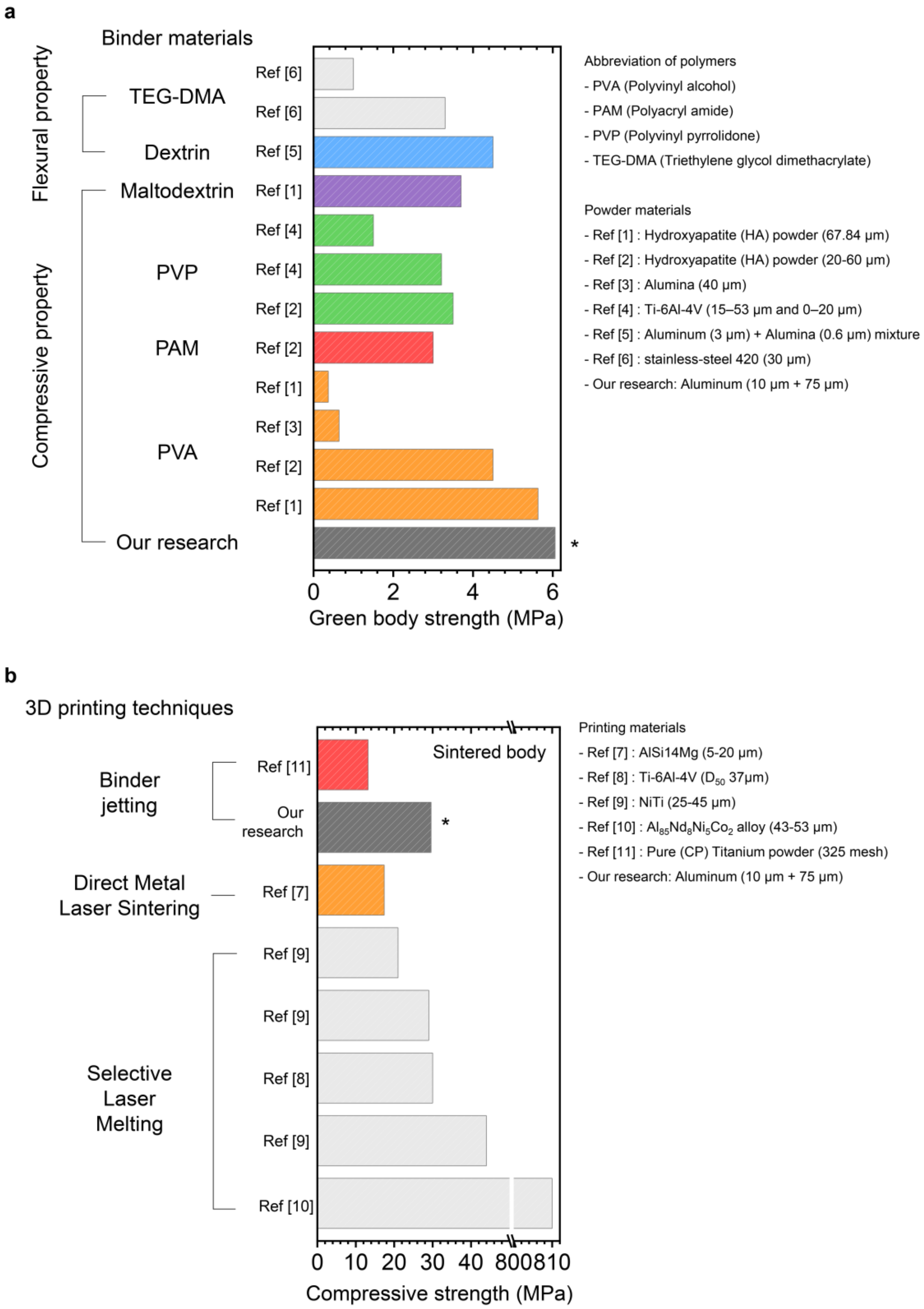

Supplementary Fig. 18. a The recent technological progress in BJM3DP techniques, with respect to the mechanical strengths of printed green bodies based on various binder materials[1-6]. **b** The recent technological progress in metal 3D printing techniques with respect to the mechanical strengths of objects[7-11]. Each study is based on different techniques as well as different metal powder such as Ti-6Al-4V and NiTi.

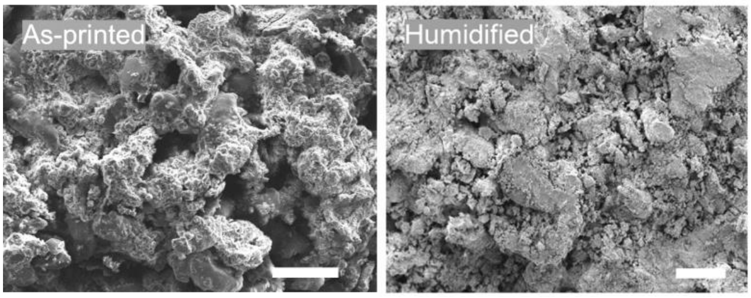

Supplementary Fig. 19. Cross-sectional SEM image of 3D-printed Al object after humidification. (scale bar: 100 μm).

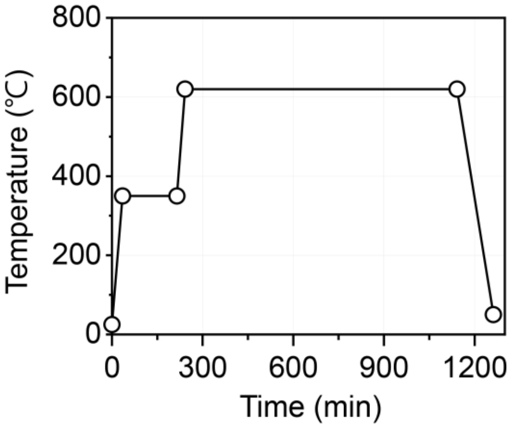

Supplementary Fig. 20. The heat treatment profile used for the debinding and sintering of Al-based 3D printed objects.

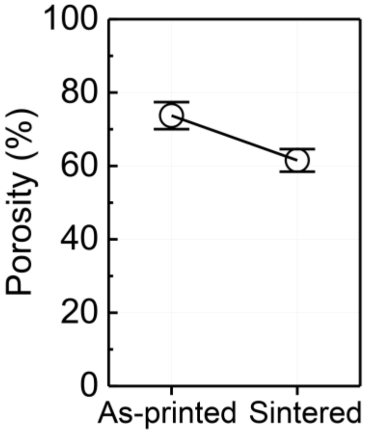

Supplementary Fig. 21. Interparticle porosities of as-printed Al objects and sintered Al objects. Data are presented as mean values ± standard deviations.

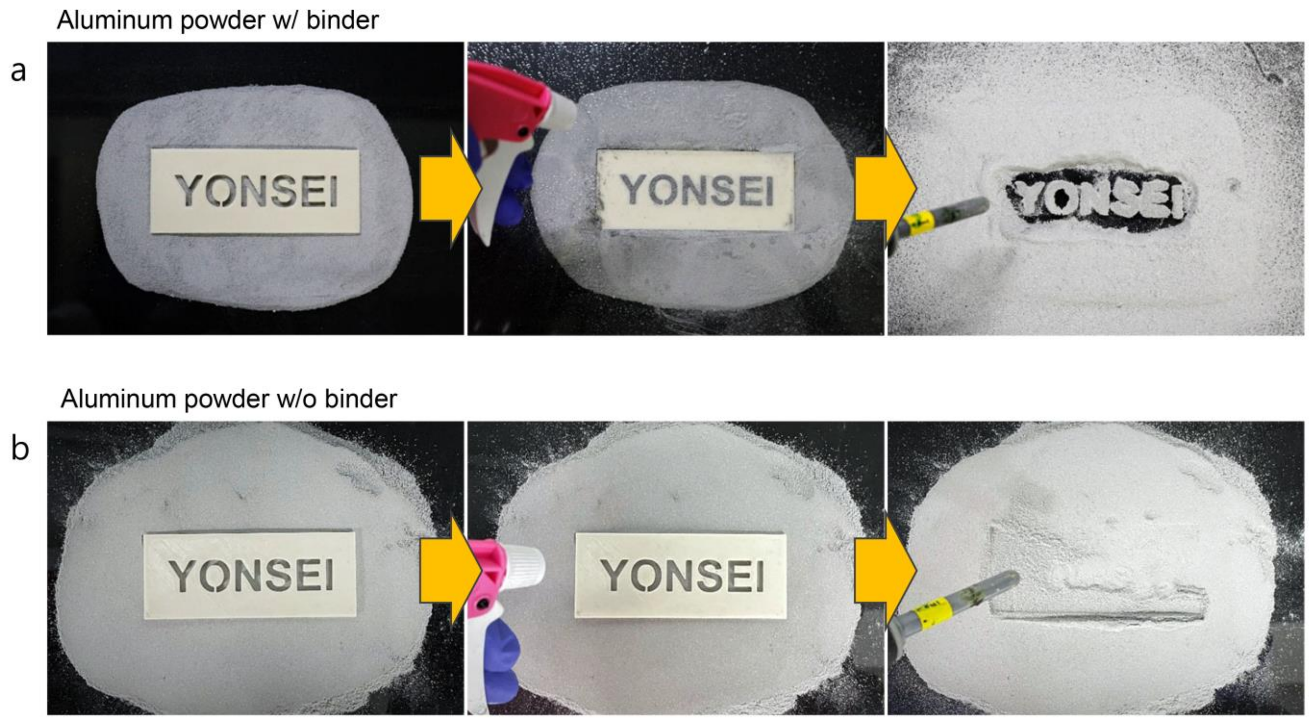

Supplementary Fig. 22. Structure formulations of Al powder upon spray water. **a** Al-NaCit system, **b** Al-only system. After the spraying water on the powder through pattern (YONSEI), the Al-only system does not maintain its pattern when dusted by an air blowgun.

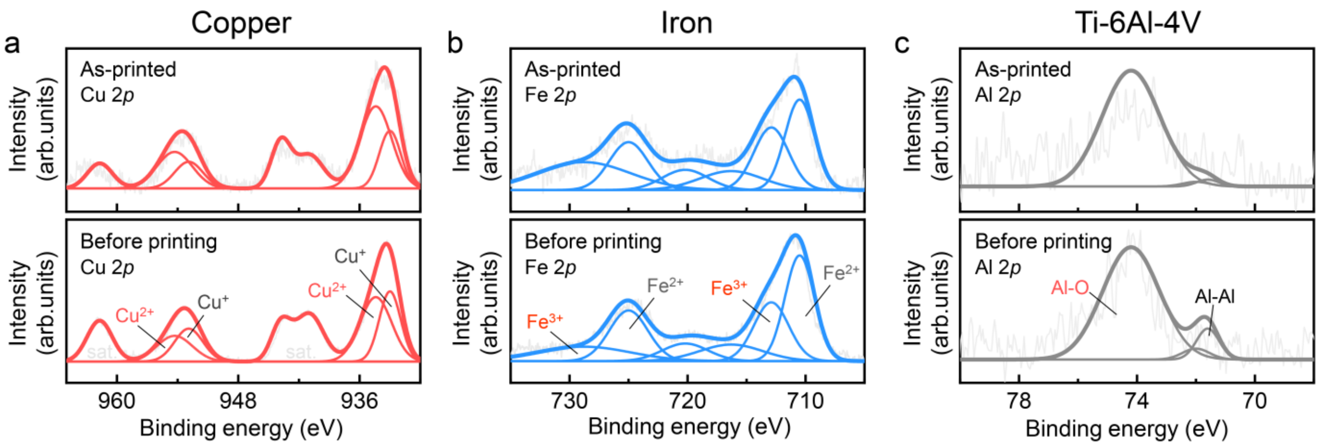

Supplementary Fig. 23. The XPS spectra of BJM3DP printed (top) and pure metals (bottom) for **a** Cu, **b** Fe and **c** Ti-6Al-4V.

Supplementary Table 1. Carboxylate asymmetric (v_{as}) and symmetric (v_s) vibrational frequencies of carboxyl-based chelators and chelated complex ($\Delta v = v_{as} - v_s$).

		v_{as} (cm^{-1})	v_s (cm^{-1})			Δv (cm^{-1})		
Chelator	NaCit	1592	-	1394	-	-	198	-
	NaTar	1622	1411	1377	1342	211	245	280
	NaSuc	1560	1443	1398	-	127	156	-
Chelated Complex	NaCit	1572	-	1392	-	-	180	-
	NaTar	1616	1405	1379	1341	211	237	375
	NaSuc	1556	1434	1404	-	122	152	-

Supplementary Table 2. Summary of 3D printing process parameters.

Parameter	Value	Unit
Powder spreading speed	1000	mm/min
Printing speed	2000	mm/min
Ink droplet size	33	pL
Printing resolution	600	DPI
Inkjet nozzle temperature	70	ºC
Builder platform temperature	70	ºC

Supplementary References

1. Zhou, Z., Lennon, A., Buchanan, F., McCarthy, H. O. & Dunne, N. Binder jetting additive manufacturing of hydroxyapatite powders: Effects of adhesives on geometrical accuracy and green compressive strength. *Addit. Manuf.* **36**, 101645 (2020).
2. Chai, W. et al. The printability of three water based polymeric binders and their effects on the properties of 3d printed hydroxyapatite bone scaffold. *Ceram. Int.* **46**, 6663-6671 (2020).
3. Kunchala, P. & Kappagantula, K. 3d printing high density ceramics using binder jetting with nanoparticle densifiers. *Mater. Des.* **155**, 443-450 (2018).
4. Tang, Y., Huang, Z., Yang, J. & Xie, Y. Enhancing the capillary force of binder-jetting printing ti6al4v and mechanical properties under high temperature sintering by mixing fine powder. *Metals* **10**, 1354 (2020).
5. Solis, D. M., Silva, A. V., Volpato, N. & Berti, L. F. Reaction-bonding of aluminum oxide processed by binder jetting. *J. Manuf. Process.* **41**, 267-272 (2019).
6. Gilmer, D. et al. An in-situ crosslinking binder for binder jet additive manufacturing. *Addit. Manuf.* **35**, 101341 (2020).
7. Yan, C. et al. Evaluation of light-weight alsi10mg periodic cellular lattice structures fabricated via direct metal laser sintering. *Journal of Materials Processing Technology* **214**, 856-864 (2014).
8. Challis, V. J. et al. High specific strength and stiffness structures produced using selective laser melting. *Materials & Design* **63**, 783-788 (2014).
9. Speirs, M., Van Hooreweder, B., Van Humbeeck, J. & Kruth, J. P. Fatigue behaviour of niti shape memory alloy scaffolds produced by slm, a unit cell design comparison. *Journal of the Mechanical Behavior of Biomedical Materials* **70**, 53-59 (2017).
10. Prashanth, K. G. et al. Production of high strength al85nd8ni5co2 alloy by selective laser melting. *Additive Manufacturing* **6**, 1-5 (2015).
11. Wiria, F. E. et al. Printing of titanium implant prototype. *Materials & Design* **31**, S101-S105 (2010).
